# Magnetic compounds with exotic Archimedean lattices

**DOI:** 10.1016/j.xinn.2025.100981

**Published:** 2025-06-05

**Authors:** Shu Guo, David A. Krug, Brianna R. Billingsley, Jianqiao Wang, Zhibin Qiu, Tai Kong

**Affiliations:** 1 Shenzhen Institute for Quantum Science and Engineering, Southern University of Science and Technology, Shenzhen 518055, China; 2 International Quantum Academy, Shenzhen 518048, China; 3 Department of Physics, University of Arizona, Tucson, AZ 85721, USA; 4 Department of Chemistry and Biochemistry, University of Arizona, Tucson, AZ 85721, USA

## Abstract

Geometrically frustrated magnetic materials provide an important platform for studying emergent quantum magnetism. Materials that host a triangular or Kagome magnetic sublattice have been intensively studied within this realm of research. Here, we point out that more lattice types can be considered geometrically frustrated since a single triangular motif is sufficient to introduce geometrical frustration. Archimedean lattices present uniform tiling in space. In addition to triangular and Kagome lattices, Archimedean lattices include maple-leaf (ML), Shastry-Sutherland (SS), trellis, ruby, and star lattices that are all triangle containing. Through a systematic search of the literature and known inorganic crystal structure databases (ICSDs), we identify materials that realize these less-common lattice types, offering new opportunities to study frustrated magnetism in diverse settings.

## Main text

Understanding magnetism in different crystalline and chemical environments has been one of the main drivers for condensed matter physics research.^1^ Recent decades’ research has shown that many emergent phases are closely related to quantum magnetism, for instance, quantum criticality, superconductivity, and quantum spin liquids (QSLs).^2^ Magnetic exchange interactions, quantum magnetic fluctuations, and orbital hybridization are some of the factors that influence the magnetic ground state. Among these factors, magnetism is also strongly affected by the geometry of the magnetic sublattice in compounds. Using a so-called geometrically frustrated lattice is one of the most effective ways to access exotic magnetic ground states due to a lack of a solution to simultaneously satisfy all magnetic interactions.^2^ Such geometrical frustration is often achieved by incorporating a triangular magnetic substructure building motif. When considering a closed loop of spins that are connected via nearest-neighbor (NN) antiferromagnetic interactions, an odd number of antiferromagnetic pairs gives rise to a competing interaction and, thus, magnetic frustration. The use of the triangular building motif yields a triangular lattice and Kagome lattice in two dimensions (2D) and pyrochlores in three dimensions (3D). Although this frustration is induced by similar triangular motifs, different lattice types present different relative energies for competing magnetic interactions and, therefore, different theoretically proposed ground states.^3^ In a triangular lattice, edge-sharing triangles give 6 NNs, as compared to the corner-sharing Kagome lattice, where only 4 NNs need to be considered in theoretical modeling. There have been many experimental searches for QSLs in real materials based on these lattice types, exploring not only QSLs but also other emergent magnetic states in the hopes of deepening our understanding of many-body entanglement physics.^3^

As geometrical frustration can be induced by just one triangle, more lattice types can host such triangular motifs mathematically. Archimedean lattices (ALs) offer a complete description of the uniform tiling in space using regular polygons where all sites of a given AL are vertex transitive. This simplicity makes ALs an ideal platform to study the geometrical influence on the magnetic ground state. ALs include both unfrustrated lattice types, like a square lattice, and frustrated types, such as triangular and Kagome lattices. Theoretically, the first systematic attempt to study the magnetic ground-state properties of ALs was given in Richter et al.,^4^ where the exact diagonalization results for the ground-state energies and order parameters in the spin ½ Heisenberg antiferromagnet model were presented. Other methods, such as the coupled-cluster method^5^ and the Monte Carlo method,^6^ were later used to investigate the magnetic state in ALs. The long-range magnetic ordering in ALs was shown to be heavily dependent on geometrical frustration, coordination number, filling level of triangles, spin number, spin model, and, moreover, the competition of nonequivalent NN bonds.^5^^,^^6^ The maple-leaf (ML) lattice has recently been predicted to be the only other lattice type, other than Shastry-Sutherland (SS), that leads to an exact dimer ground state.^7^ Certainly, theoretical investigation within the triangular lattice alone would produce various ground states based on details in the theoretical model, let alone 11 different ALs. The inclusion of this additional geometrical tuning factor in ALs will further facilitate the discovery of more emergent magnetic ground states.

Most experimental investigations on geometrical frustration to date follow theoretical guidelines on triangular or Kagome lattice types. This perspective aims to identify possible real magnetic material systems that exhibit other, by-far missed, geometrically frustrated lattice types, namely ML, SS, trellis, ruby (also known as a bounce lattice), and star lattices. The survey includes both manual and automated searches. The manual search was done via a keyword search in the literature. To thoroughly identify existing compounds that could be suitable for ALs’ magnetism studies, an automated search using crystallographic information and lattice matching tools is highly efficient. The community benefited from such high-throughput computational methods to look for compounds that exhibit certain crystalline characteristics, such as a Kagome lattice.^8^ Our automated search utilizes an open-source database, the Materials Project.^9^ The primary goal of this search is to find magnets that show magnetism that are due only to ALs. Therefore, materials containing one and only one of common magnetic 3d transition metal elements (Ti-Cu) and magnetic rare-earth elements (Ce-Nd and Sm-Yb) were used for analysis. A total of 51,340 compounds were downloaded from the Materials Project. The Systre program was used to identify the lattice type of magnetic elements. Systre uses a vector representation of periodic graphs and a barycentric equilibrium placement method to generate a “key” for each type of lattice net.^10^ It addresses the problem of lattice distortions found in real materials via the identification and computation of the combinatorial symmetry of a crystal net. It has been used, for example, in metal-organic-framework research^11^ and a recent survey for flat-band materials.^12^ The associated reticular chemistry structure resource (RCSR) contains hundreds of lattice net types in different dimensions, including ALs that are of interest in this survey. The corresponding symbols in RCSR for each exotic AL are *fsz* (ML), *tts* (SS), *cem* (trellis), *htb* (ruby), and *hca* (star). When a magnetic atom occupies more than one atomic site in the crystal structure, all atomic sites associated with the magnetic atom were treated as one net in Systre so that only the simplest nets were kept. Site occupancy and disorder were not considered in the search. The output from Systre was then manually screened to ensure the final lattice type could be associated with a certain AL in 2D. This step is important, for example, to reduce the trellis lattice results from a few thousand due to its similarity to the square lattice to a few hundred, which were finally reported here. Compounds are then further grouped according to their crystal structural types to illustrate the chemical tunability and variety of certain magnetic systems. The automated search was able to identify most of the candidate materials that were found through manual searches. Compounds with their ICSD numbers missing in the Materials Project, such as listed star lattice compounds, and compounds that are different but closely related, such as ZnMn_3_O_7_·3H_2_O and Na_2_Mn_3_O_7_ in the ML, benefit from manual search additions. Detailed results with representative compounds in each structure type are shown in Table 1.

**Table 1:** Lattice types with corresponding representative material candidates

Maple-leaf (3^4^,6)	Bounce/Ruby (3,4,6,4)	Shastry-Sutherland (3^2^,4,3,4)	Trellis (3^3^,4^2^)	Star (3,12^2^)
				
**Na**_**2**_Mn_3_O_7_ (ICSD: 92858)**Zn**Mn_3_O_7_·3H_2_O (ICSD: 202701)Bi_4_Mn_3_O_11.5_ (ICSD: 424652)Pb_3_Cu_6_TeO_6_(OH)_7_Cl_5_ (2 ICSD: 290104)Cu_6_TeO_4_(OH)_9_Cl (ICSD: 241339)Cu_6_IO_3_(OH)_10_Cl (ICSD: 241338)Cu_2_ZnAsO_4_(OH)_3_ (ICSD: 0006608)Cu_6_**Al**(SO_4_)(OH)_12_Cl_3_·3H_2_O (ICSD: 73424)**K**_x_[Fe(O_2_CCH_2_)_2_NCH_2_PO_3_]_6_·nH_2_O (CCDC: 279858)	CsBa**Cr**_3_F_12_ (ICSD: 67209)**Y**Ni_2_**Al**_3_ type (ICSD: 54425)Ba**Eu**_6_(Si_3_B_6_O_24_)(OH)_2_ (ICSD: 194860)	**AlCu**_2_**Re**_2_ (ICSD: 57706)**Ti**_2_**ReB**_2_ (ICSD: 44554)Zr_4_CuSb_7_ (ICSD: 195056)Li_2_Ca(MnN)_2_ (ICSD: 411203)Ca_5_Cd**Cu**_2_ (ICSD: 423251)BaCa_4_**Cu**_2_N_4_ (ICSD: 86066)**Co**[PH_2_O_2_]Cl[H_2_O] (ICSD: 69561)**ErAlGe**O_5_ (ICSD: 171495)Ba**Nd**_2_**ZnS**_5_ (ICSD: 93714)**Tm**B_4_ (ICSD: 109209)**Yb**_2_**Ru**_3_Ga_10_ (ICSD: 107022)**CaCo**_2_**Al**_8_ (ICSD: 57533)**Eu**_2_SiCl_2_O_3_ (ICSD: 400126)**Dy**_2_Be_2_**Si**O_7_ (ICSD: 126614)H_2_CoP_3_O_10_[NH_3_]_3_ (ICSD: 15552)	**LiV**_2_O_5_ (ICSD: 175737)**Ca**V_2_O_5_ (ICSD: 82689)Ce_2_Te_7_**Br**_2_O_17_ (ICSD: 250636)**CeTl**_2_P_2_S_7_ (ICSD: 171219)**Eu**_2_B_4_Rh_5_ (ICSD: 68540)**Gd**_2_Ge(BO_4_)_2_ (ICSD: 168967)**Gd**_2_B_3_C_2_ (ICSD: 67980)**PrSn**_2_ (ICSD: 657389)**Sm**_2_(CN_2_)_3_ (ICSD: 240312)CaFeO_2_Cl (ICSD: 96556)	[Fe_3_(μ_3_-O)(μ-OAc)_6_(H_2_O)_3_][Fe_3_(μ_3_-O)(μ-OAc)_7.5_]_2_·7H_2_O (CCDC: 643841)[(CH_3_)_2_(NH_2_)]_3_[Cu^II^_3_(μ_3_-OH)(μ_3_-SO_4_)(μ_3_-SO_4_)_3_]·0.24H_2_O (CCDC: 1977730)

The notation (3,4,6,4) for the ruby lattice, for instance, means that every vertex is surrounded by a triangle, a square, a hexagon, and then a square. Elements that exhibit the lattice type in the column are underlined. Bold atoms can be replaced by other elements within similar structural types. Numbers in the parenthesis after each compound are ICSD or Cambridge Crystallographic Data Center (CCDC) numbers that can be used to find the corresponding crystal structure and structure type.

There are compounds found for each of the listed exotic ALs. Many of the ML and star lattice compounds and some of the SS compounds have been studied with consideration of their corresponding AL net. Most of the listed materials are yet to be studied with respect to their lattice type, which calls for more experimental investigations in the future. Despite many searches, the material candidates for ruby and star lattices are not as abundant compared to other lattice types. More material discovery will be useful in pushing forward the physical properties research in these lattice types. Although not the focus of this perspective, metal-organic frameworks can also serve as a promising direction to study geometrical frustration in these exotic ALs.^13^ With the list of compounds, a few caveats and future directions should be mentioned here.

The geometrical frustration argument comes primarily from the real space geometry of the magnetic sublattice in a compound. The actual frustrated magnetism, however, depends on the magnetic interaction between magnetic atoms in solids. The strength of a magnetic interaction is not directly related to the real space distance between atoms. In metals, the long-range interaction follows a Friedel oscillation that complicates the sign and magnitude of the interaction with respect to interatomic distance. The long-range nature also requires consideration of exchange interactions beyond the NN. In semiconductors, superexchange interaction varies with the relative angle between magnetic and nonmagnetic neighboring atoms. This angle dependency causes some trellis lattices to be better described as a spin ladder system, where the exchange interaction between square chains is much weaker than that within squares.^14^ Yet, from the material’s perspective, the magnetic lattice type is the only information that helps preselect candidate materials that can potentially realize relevant physics. In addition, although the ALs discussed here are 2D lattices, interlayer magnetic interactions in real materials—strongly influenced by the interlayer spacing and exchange pathways—play an important role in determining the magnetic ground state. Detailed physical property characterizations are needed to further confirm an ideal candidate material.

ALs are well-defined lattice nets. Real materials almost always present distortions. Distortion may not be discouraging from the argument of exchange interaction strength above. Distortion, however, will affect the identification of geometry. Famously, an SS lattice is derived from a distorted square lattice. A trellis lattice can also be achieved by applying a small shear on a square lattice or compressing a honeycomb lattice. Whereas this interconnection between lattice types adds difficulty in identifying the relevant physics models, it does provide an opportunity to tune magnetism from one model to another via tuning parameters, such as pressure or chemical doping.

Although, in this perspective, the magnetic lattice identification combines different atomic sites for magnetic elements, the discovery of new compounds may be achieved by putting a magnetic element on a known lattice site that exhibits promising lattice nets. For instance, Bi in Bi_14_Rh_3_I_9_ (ICSD: 1770743), the Co2 site in the Hf_2_Co_4_P_3_ (ICSD: 25758), the Rh2 and Ge2 sites in LaRh_6_Ge_4_ (ICSD: 426482), the Al2 site in SrCo_2_Al_9_ (ICSD: 57633), and the Si site in Ce_2_CoSi_3_ (ICSD: 83895) form the ruby lattice. Compounds that crystallize in these structures may be modified to serve as platforms to study magnetism in a ruby lattice. It is also worth mentioning that the list of magnetic elements in the search can be non-moment bearing. One example is that Ni in YNi_2_Al_3_ does not bear a local moment.^15^ The magnetism of elements in solids depends on their oxidation state and conductive environment, which needs to be determined experimentally. Compounds like YNi_2_Al_3_ are still of interest because other variations within the same structural family may still have the potential to host quantum magnetism behaviors.

To conclude, the authors point out that more material systems can be considered when studying geometrical frustration. Candidate materials that exhibit less common ALs, namely the ML, SS, trellis, ruby, and star lattices, have been identified via a thorough search of the existing literature as well as the known inorganic compound database. The listed materials are worth further experimental investigation under the realm of the corresponding quantum magnetism model for each lattice type.

## Funding and acknowledgments

The work at the University of Arizona was supported by the US Department of Energy (DOE), Office of Science, Basic Energy Sciences (BES), under award DE-SC0025301. B.R.B. acknowledges support from the 10.13039/100000001National Science Foundation under award DGE-2137419. S.G., J.W., and Z.Q. acknowledge the financial support from the 10.13039/501100001809National Natural Science Foundation of China (22205091). B.R.B. acknowledges support from the National Science Foundation under award DGE-2137419. We would like to thank Zhongai Li for technical support. The funders had no role in the study design, data collection and analysis, decision to publish, or preparation of the manuscript.

## Declaration of interests

The authors declare no conflicts of interest.
